# Nanoliposomes as Effective Vehicles of Antioxidant Compounds in Food and Health

**DOI:** 10.3390/ijms26125523

**Published:** 2025-06-09

**Authors:** Jonathan García-Morales, Diana Fimbres-Olivarría, Ricardo Iván González-Vega, Ariadna Thalía Bernal-Mercado, Santiago Pedro Aubourg-Martínez, Karla Alejandra López-Gastélum, Miguel Ángel Robles-García, José de Jesús Ornelas-Paz, Saúl Ruiz-Cruz, Carmen Lizette Del-Toro-Sánchez

**Affiliations:** 1 Departamento de Investigación y Posgrado en Alimentos, Universidad de Sonora, Blvd Luis Encinas & Rosales S/N, Hermosillo 83000, Sonora, Mexico; thalia.bernal@unison.mx (A.T.B.-M.); karla.lopezgastelum@unison.mx (K.A.L.-G.); saul.ruizcruz@unison.mx (S.R.-C.); 2 Departamento de Investigaciones Científicas y Tecnológicas, Universidad de Sonora, Blvd Luis Donaldo Colosio S/N, Hermosillo 83000, Sonora, Mexico; 3 Departamento de Ciencias de la Salud, Centro Universitario de los Valles, Universidad de Guadalajara, Carr. A Guadalajara Km 45.5, Ameca 46600, Jalisco, Mexico; ricardo.gonzalez@academicos.udg.mx; 4 Departamento de Tecnología de Alimentos, Instituto de Investigaciones Marinas, Calle Eduardo Cabello No. 6, Vigo, C.P. 36208 Ponevedra, Spain; saubourg@iim.csic.es; 5 Departamento de Ciencias Médicas y de la Vida, Centro Universitario de la Ciénega, Universidad de Guadalajara, Av. Universidad 1115, Col. Lindavista, Ocotlán 47820, Jalisco, Mexico; miguel.robles@academicos.udg.mx; 6 Centro de Investigación en Alimentación y Desarrollo A.C.-Unidad Cuauhtémoc, Av. Río Conchos S/N, Parque Industrial, Ciudad Cuauhtémoc 31570, Chihuahua, Mexico; jornelas@ciad.mx

**Keywords:** nanoliposomes, encapsulation, antioxidant compounds, affinity, human health

## Abstract

Nanoliposomes have increased exponentially since their discovery in the 1960s, primarily for encapsulating medicines or compounds that can improve human health. However, recent studies propose nanoliposomes as vehicles to protect, transport, and subsequently release compounds of various kinds to fortify the properties of foods and cause a prolonged release of encapsulated substances in a specific part of the body. Among the compounds successfully encapsulated are β-carotene; α-carotene; vitamins A, C, and D; and lycopene, among others. The encapsulation of extracts with high contents of antioxidant pigments is still to be explored. Therefore, this review aims to compile the compounds that have been successfully encapsulated and have met the specific prolonged release criteria, highlighting areas of research opportunity and application such as biomedical, pharmaceutical, and nutraceutical industries.

## 1. Introduction

Nanoliposomes or nanoliposomal vehicles are molecules composed of a double membrane of phospholipids organized in a lipid bilayer: the polar ends are in the center, trapping a small volume of the aqueous phase, while the apolar tails are arranged outward and interact with the lipophilic ends of the external phospholipids, which position their polar heads outward. This creates a molecule capable of storing lipophilic and hydrophilic compounds [1,2].

Nanoliposomes’ encapsulation efficiency (EE) is higher than those with exclusively hydrophilic properties because the encapsulated substances remain firmly bound to the lipophilic tails [3]. Among the bioactive materials successfully encapsulated are genetic material, proteins, DNA, peptides, vaccines, and enzymes, as well as anticancer, antimicrobial, antioxidant, antihemolytic, and anti-inflammatory agents [4].

Recent nanoliposome research has focused on adding them to foods to enhance and fortify the product’s properties for consumption. Factors determining a successful encapsulation and specific prolonged release include encapsulation efficiency, process yield, prolonged release factor, particle size, and Z potential [4].

On the other hand, consuming antioxidants and natural products is one of the most addressed issues today, as the population is becoming more health-conscious. However, it is not just about consumption but the absorption of molecules that provide antioxidants for their biological activity. Beyond protecting pigments from enzymatic and acidic degradation in the digestive system, nanoliposomes aim to ensure the arrival and subsequent release of pigments into the intestine, where the absorption process occurs [5].

One of the current problems in society is people’s concern regarding the consumption of functional foods. Due to the current high rates of development of chronic–degenerative diseases such as leukemia, blindness, osteoarthritis, diabetes, cardio-cerebrovascular diseases, and various types of cancer [6], food research has chosen to explore the encapsulation of compounds with antioxidant, anti-inflammatory, antimicrobial, antihemolytic, medicinal, and analgesic properties in vesicles formed by phospholipids (nanoliposomes).

Therefore, the objective of this review is to compile compounds with antioxidant, anti-inflammatory, antihemolytic, or photoprotective properties suitable for nano-encapsulation in liposomes, their application in foods, and their impact on health, as well as to provide a perspective on unexplored areas and prospects for extracts of different sources. Although this review focuses on nanoliposomes, references to other nanosystems, such as nanoemulsions, various types of nanoparticles, and nanostructured lipid carriers, are included where relevant. These comparisons aim to contextualize nanoliposomes’ advantages, limitations, and future outlook in food and health applications.

## 2. Nanoliposomes

### 2.1. Structure and Properties of Nanoliposomes

Various delivery systems, such as nanoemulsions, microemulsions, lipid nanoparticles, biopolymeric nanoparticles, and liposomes, have been extensively studied in the field of nanotechnology. These systems are primarily explored for their potential to encapsulate and enable bioactive compounds’ prolonged release, particularly in developing functional foods [7]. Specifically, liposomes are spherical vesicles composed of one or more phospholipids arranged in bilayers or multilayers enclosing an aqueous core. They range from nanometers to micrometers (20–100 µm). When their diameter is below 100 nm, they are specifically referred to as nanoliposomes. Nanoliposomes possess distinct physicochemical and biological properties compared to conventional (larger) liposomes, such as improved cellular uptake, enhanced stability, and better penetration into biological barriers, making them especially attractive as carriers in food and pharmaceutical applications [8].

**Figure 1:**
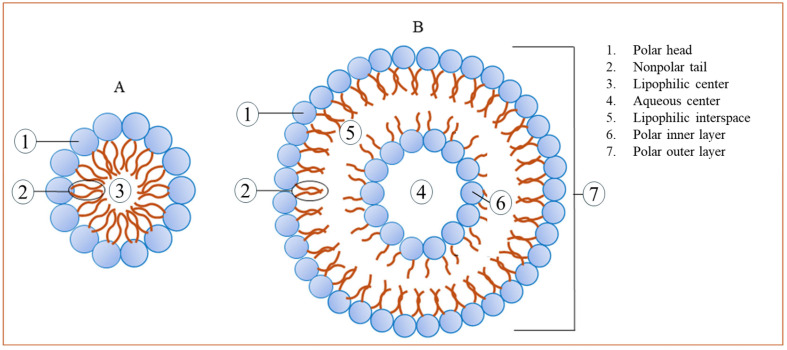
Comparative scheme between a micelle (**A**) and a nanoliposome (**B**). In aqueous environments, micelles are formed from amphiphilic molecules, creating a spherical structure with a hydrophobic core and hydrophilic exterior, ideal for encapsulating hydrophobic compounds. In contrast, liposomes consist of one or more phospholipid bilayers, encapsulating hydrophilic substances (in the aqueous center, no. 4) and hydrophobic substances (within the bilayer, no. 5).

Phospholipids, the building block of liposomes, consist of a polar (hydrophilic) head and nonpolar (hydrophobic) tails. In an aqueous environment, these molecules arrange to form a core where hydrophilic compounds can be encapsulated (aqueous center). Additionally, the nonpolar tails interact with the nonpolar tails of other phospholipids, forming an intermediate layer in which lipophilic compounds can be encapsulated (lipophilic interspace). Finally, the polar heads of the outer phospholipids complete the spherical structure (Figure 1) [1,2]. The head determines the surface charge of the liposome (neutral, cationic, or anionic) and is generally composed of choline, phosphate, and glycerol. The hydrophobic tail comprises one or two fatty acid chains of 14–18 carbons that can be saturated or unsaturated hydrocarbon chains and varying degrees of stiffness and permeability [9].

Phospholipids used in the production of liposomes and nanoliposomes can be of natural origin or synthetic [10]. Natural phospholipids can be obtained from vegetable sources such as soybeans, sunflower, wheat germ, rape (canola) seed, flax seed, and animal material, like egg yolk or milk. They are generally biocompatible and suitable for food applications, but their lipid composition is less controlled. Synthetics are prepared with well-defined phospholipids produced through chemical synthesis. Some of the synthetic phospholipids used are dimyristoyl, dipalmitoyl, or distearoylphosphatidylcholine. Synthetic phospholipids are homogeneous regarding the polar head group and fatty acid structure, which offer improved reproducibility, better control over bilayer properties, and allow for the design of liposomes with specific rigidity or permeability. In addition, cationic phospholipids or anionic phospholipids such as phosphatidic acid or phosphatidylserine can give liposomes a net surface charge. Liposomes’ surface charge can determine how they interact with tissues and cells. On the other hand, modifying the liposome surface can improve specific characteristics, such as immunocompatibility. For example, incorporating polyethylene glycol (PEG) chains on their surface by conjugating PEG-lipids (e.g., DSPE-PEG2000) into the bilayer imparts a “stealth” character, reducing recognition and clearance by the mononuclear phagocyte system, and significantly prolongs circulation time in vivo [11]. Although commonly used in pharmaceutical formulations, PEGylation is also being explored in food-grade nanocarriers for enhanced stability and targeted delivery. The most frequently used phospholipids in liposome preparation are shown in Table 1.

In addition to phospholipids, liposomes may also contain sterols in their structure. The most widely used sterol in liposome production is cholesterol. Cholesterol provides physical and biological stability, as it can modify the viscosity or rigidity of the bilayer while reducing its permeability in the presence of biological fluids such as blood. In the absence of cholesterol, the bilayer could experience rupture [12,13]. Cholesterol can be integrated into phospholipid membranes at high concentrations, with molar ratios of 1:1 or 2:1 relative to phospholipids. The appropriate amount of cholesterol in nanoliposomal compositions is determined mainly according to the potential application. Lipid composition and cholesterol concentration are essential in designing and developing functional nanoliposome systems [8].

**Table 1:** Most used phospholipids in liposome formation.

Natural	Synthetic	Function	Reference
Phosphatidylcholine (PC) 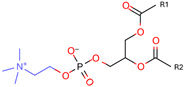	1,2-dimyristoyl-sn-glycero-3-phosphocholine (DMPC) 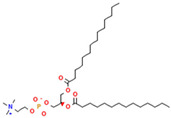	Increase membrane fluidity and eicosanoid production	[14]
Phosphatidylethanolamine (PE) 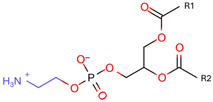	1,2-dioleoyl-sn-glycero-3-phosphocholine (DOPC) 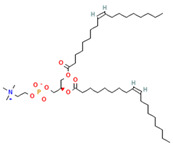	PC precursor promotes membrane fusion, oxidative phosphorylation, and mitochondrial biogenesis	[15]
Phosphatidylserine (PS) 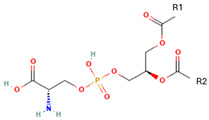	1,2-Distearoyl-sn-glycero-3-phosphocholine (DSPC) 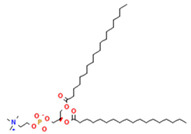	PE decarboxylation, autophagosomes formation, morphology regulation and dynamics and functions of mitochondria	[16]
Phosphatidylglycerol (PG) 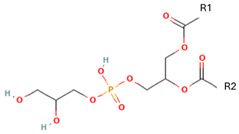	1,2-Dipalmitoyl-sn-glycero-3-phosphoglycerol (DPPG) 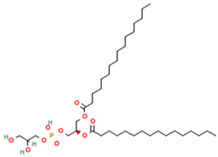	Important role in apoptosis and blood clotting, besides serving as a conduit for the transfer of lipids between organelles	[17]
Phosphatidylinositol (PI) 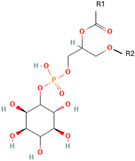	1,2-Distearoyl-sn-glycero-3-phosphoglycerol (DSPG) 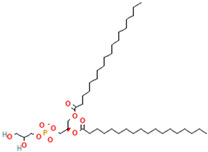	Regulates traffic to and from Golgi apparatus and helps protect against hepatic viruses	[18]
Phosphatidic acid (PA) 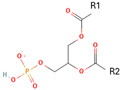		Serve as a fusogenic lipid, altering membrane structure and promoting membrane fusion, especially in neurons	[19]

R1 and R1 = alkyl chains derived from fatty acids, commonly ranging from 14 to 22 carbon atoms.

### 2.2. Preparation of Nanoliposomes

**Figure 2:**
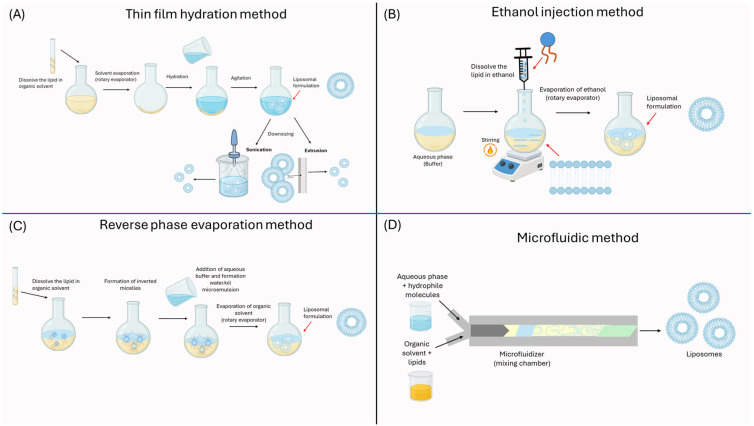
Representative schemes of the four used methods for the preparation of nanoliposomes. This figure illustrates the core steps involved in (**A**) the thin-film hydration method, (**B**) ethanol injection, (**C**) reverse-phase evaporation, and (**D**) the microfluidic method.

Liposomes and nanoliposomes do not form spontaneously; additional energy is required to overcome the system’s energetic barrier. Lipid vesicles occur when phospholipids are dispersed in an aqueous environment, but only if enough energy is provided to reorganize lipid molecules into bilayer vesicles, resulting in thermodynamic stability in the aqueous medium. Liposomes and nanoliposomes are created with various physical, chemical, and technological considerations, such as lipid composition, encapsulated component characteristics, vesicle size, stability, and scalability. Many strategies have been developed, each with unique advantages and limits. The review by Lombardo and Kiselev [20] provides an exhaustive and detailed analysis of the various methods used to prepare nanoliposomes, including illustrative schemes for each method; therefore, only the most essential characteristics of these techniques will be summarized here. Figure 2 shows some of the most used methods for liposomes production.

The thin-film hydration technique is the classical and most extensively utilized technique to produce liposomes and nanoliposomes. This process begins with dissolving phospholipids in volatile organic solvents such as chloroform or methanol. Controlled evaporation of the solvent using a rotary evaporator generates a thin lipid film adhering to the walls of the flask. Subsequent hydration of this film with an aqueous solution at temperatures above the lipid phase transition (generally 50–60 °C) induces spontaneous self-assembly into multilamellar vesicles. These can be transformed into tiny unilamellar vesicles using size-reduction procedures such as probe sonication or extrusion. Although this method offers good encapsulation of hydrophilic compounds (up to 30% efficiency), it has limitations in industrial scalability due to large volumes of organic solvents and long processing times [20,21].

Solvent injection methods include rapidly diluting lipids dissolved in a water-miscible organic solvent into an aqueous phase. Because of its ease of use, the ethanol injection method is commonly employed to prepare liposomes and nanoliposomes. When a lipid-ethanol solution is introduced into an aqueous buffer and stirred, the lipids self-assemble into vesicles due to fast solvent diffusion. The approach typically produces small unilamellar vesicles with sizes ranging from 30 to 100 nm. However, particle size control is limited, and leftover solvents may be a concern for medicinal or food applications [22].

The reverse phase evaporation method is very successful at encapsulating hydrophilic compounds. Lipids dissolved in a chloroform and diethyl ether mixture are emulsified with an aqueous phase containing the active chemical to generate a water-in-oil emulsion frequently sonicated with probes. When the organic solvents are removed under reduced pressure, the lipid gel collapses, forming large unilamellar vesicles (LUVs). Reverse-phase evaporation often has better encapsulation efficiency than hydration techniques. However, organic solvents and the potential exposure of delicate molecules to sonication and heat are significant disadvantages [20,23].

The detergent removal method uses detergents to solubilize lipids and create mixed micelles. When the detergent is gradually removed via dialysis, gel filtration, or adsorption onto polystyrene beads, the lipids reorganize into liposomal bilayers. This approach is particularly useful for reassembling membrane proteins into liposomes because it allows for delicate insertion into the bilayer. Nonetheless, the low encapsulation efficiency for hydrophilic compounds and the complexity of detergent removal limit its widespread application [24].

On the other hand, size reduction is frequently required to attain nanometric dimensions with a consistent size distribution. Two major strategies are used: sonication and extrusion. Using either a probe or bath sonication, sonication uses ultrasonic radiation to break apart prominent vesicles into small vesicles. While efficient for manufacturing nanosized liposomes (<100 nm), it may degrade sensitive molecules due to localized heating and shear stresses. With the extrusion, liposomes are forced through polycarbonate membranes with predetermined pore diameters using pressure or nitrogen. Multiple cycles produce monodispersed nanoliposomes with remarkable repeatability [20,25].

Modern strategies have been developed to overcome the limits in scalability and reproducibility of traditional methods. High-pressure homogenization reduces the size of prepared vesicles through mechanical shear forces and cavitation [26]. It is ideal for industrial-scale production and is widely used in food and cosmetic applications. However, the size distribution may be larger, and encapsulation efficiency may be reduced during processing. Microfluidic mixing is a highly regulated, continuous process for mixing lipids and aqueous phases on the microscale. Rapid and uniform mixing allows for the creation of small unilamellar vesicles with precise size control (usually 50–150 nm) and low polydispersity [8]. This technology is scalable and compatible with the manufacture of injectable nanoliposome formulations.

Each technique varies in cost, complexity, and regulatory compliance, particularly when moving from bench-scale to industrial-scale manufacturing. The optimal procedure is determined by the desired use (pharmaceutical, cosmetic, or food-grade) and the physicochemical qualities of the active chemicals to be encapsulated.

Sterilization is a crucial step in the development of nanoliposome-based formulations for biomedical, pharmaceutical, and food applications. However, this process presents specific challenges due to their nanometric size, lipidic nature, and structural sensitivity [27]. Conventional sterilization methods may damage vesicle integrity, cause aggregation, destroy encapsulated bioactive compounds, or alter the physicochemical properties of the lipid bilayer. Thus, choosing an appropriate sterilization procedure necessitates balancing microbiological safety with formulation stability and usefulness. Among the conventional sterilization techniques, heat treatments are effective for eliminating microbial contamination; however, they are generally not suitable for nanoliposomes due to the risk of bilayer disruption, drug leakage, or lipid hydrolysis, particularly in formulations containing unsaturated phospholipids or thermolabile compounds [27]. Non-thermal methods, such as gamma and ultraviolet irradiation, have been explored in liposomal vaccines and parenteral formulations, as well as in aqueous liposomal dispersions. Nevertheless, this method can cause ionizing radiation, inducing lipid peroxidation, free radical formation, potential photodegradation, and degradation of encapsulated actives, particularly antioxidants or sensitive peptides. Furthermore, in the case of UV sterilization, it is limited by its low penetration depth. Although ethylene oxide gas sterilization is successful and commonly used in medical device manufacturing, it is unsuitable for nanoliposomes because it can leave hazardous residues and interact negatively with lipid components [27,28]. Sterile membrane filtration is the most often used approach for nanoliposomes, especially those with particle sizes less than 200 nm. The formulation is passed through a 0.22 µm pore filter under aseptic conditions. This approach protects liposomal integrity while avoiding thermal or chemical destruction. However, it is limited by filter clogging, particularly in viscous or polydisperse systems, as well as liposome retention at the cutoff. As a result, careful control over liposome size distribution and polydispersity is required prior to filtration [29]. Currently, research is actively exploring alternative sterilization methods. In this context, supercritical carbon dioxide (ScCO_2_) has emerged as a promising strategy for sterilizing sensitive products, including liposomes [30]. ScCO_2_ offers several advantages: it is environmentally friendly, non-flammable, non-toxic, chemically inert, physiologically safe, and cost-effective. Moreover, it enables sterilization at low temperatures, making it particularly suitable for temperature-sensitive formulations [30].

### 2.3. Stability of Nanoliposomes

Nanoliposome stability is important in determining their functionality, mainly when used in food, medicinal, or cosmetic compositions. One of the most challenging issues is maintaining the physical stability of liposomal suspension, which is frequently disrupted by lipid vesicle aggregation, fusion, or coalescence [31]. These activities limit functional surface area and may result in the premature release of encapsulated bioactive chemicals, affecting efficacy and shelf life. In this situation, instability is typically related to nanoliposome self-aggregation or vesicle fusion, which results in increased particle size and decreased homogeneity. This coalescence effect must be scrupulously avoided because it affects the formulation’s physicochemical qualities and diminishes its efficacy in protecting and delivering active components [32]. To characterize how long nanoliposome formulations retain their stability, physicochemical integrity, and functional performance, particle size distribution measurements, zeta potential, encapsulation efficiency, and membrane integrity are made over time [33].

Lipid composition has a significant impact on both encapsulation efficiency and stability. Unsaturated phospholipids increase membrane fluidity and may facilitate the encapsulation of hydrophilic molecules; however, they are more susceptible to oxidative degradation. Oxidative degradation mechanisms, such as ester bond hydrolysis and unsaturated fatty acid chain peroxidation, compromise the phospholipid bilayer’s structural integrity, resulting in the leakage of encapsulated substances. Lipid oxidation occurs during processing or storage and is enhanced by light, heat, oxygen, or trace metals. This process causes sensory and structural qualities to deteriorate, leading to changes in texture, color, and flavor, and the generation of cytotoxic or pro-inflammatory by-products that pose safety risks to consumers [34].

Beyond oxidative breakdown, achieving high encapsulation efficiency presents considerable hurdles, particularly for hydrophilic chemicals, which are frequently poorly preserved in the aqueous core of liposomes [35]. Lipophilic compounds integrate more easily into the lipid bilayer but may destabilize it when present in high concentration. Processing techniques also have an essential influence. High-energy methods, such as sonication, high-pressure homogenization, or extrusion, can reduce vesicle size and polydispersity, but they can also damage sensitive chemicals or threaten bilayer integrity if not adequately tuned. For example, excessive sonication can cause encapsulated drug leakage or bioactive denaturation [34].

Overall, preserving nanoliposomes’ colloidal, chemical, and oxidative stability is critical for maintaining their activity during manufacture, storage, and application. The design of strong lipid bilayers and precise control of environmental parameters remain critical to producing stable nanoliposomal systems for the food and health industries.

### 2.4. Nanoliposome as Bioactive Compound Delivery

Nanoliposomes are highly adaptable carriers that increase the stability, bioavailability, and transport of bioactive substances. They are perfect for use in medications, cosmetics, and functional foods because of their capacity to encapsulate both hydrophilic and lipophilic compounds, as well as their biocompatibility and structural resemblance to biological membranes. Numerous variables—such as the lipid composition, vesicle size, lamellarity, and the physicochemical makeup of the target environment (changes in pH, temperature, light, ionic strength, or osmotic pressure)—influence the release of encapsulated drugs from nanoliposomes [36,37].

**Figure 3:**
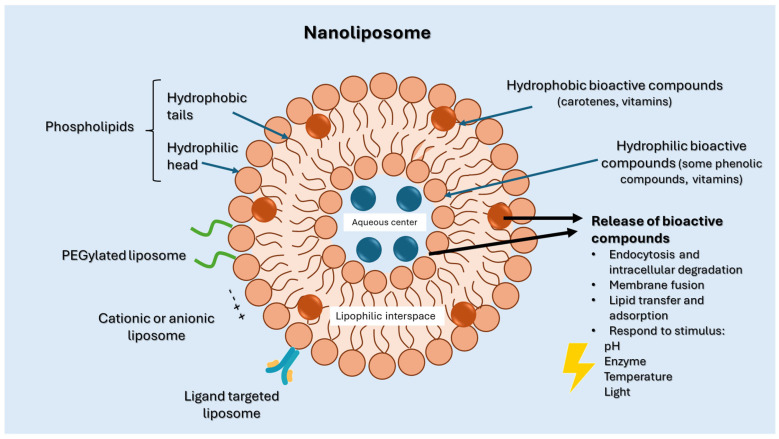
Release of bioactive compounds from nanoliposomes governed by several physicochemical and environmental factors, including the compound’s localization within the liposome, lipid composition, and external stimuli. Compounds encapsulated in nanoliposomes can be found in the aqueous core (hydrophilic molecules) or within the lipid bilayer (hydrophobic molecules).

Bioactive compounds can become trapped in the liposome’s lipid bilayer (lipophilic) or aqueous core (hydrophilic) (Figure 3). This localization directly impacts the release kinetics. Whereas lipophilic substances are usually released through lipid exchange or partitioning, hydrophilic compounds must diffuse across the bilayer or break the membrane. The fluidity of the bilayer, which is influenced by the kind of lipid, degree of saturation, and cholesterol level, influences permeability and release rate [38].

One of the most common mechanisms of bioactive compound release is cellular endocytosis, in which nanoliposomes are absorbed by target cells via energy-dependent processes. Once within the cell, the vesicles are transported to endosomes and then to lysosomes, where the acidic pH and hydrolytic enzymes aid in the breakdown of the lipid bilayer. This breakdown results in releasing the contained chemicals into the cytoplasm [39]. While effective, this route has one possible limitation: the encapsulated substance may be exposed to degradative enzymes, which can harm labile molecules unless protected or activated using prodrug techniques. Another key process is direct membrane fusion, in which the nanoliposome’s lipid bilayer merges with the cellular membrane, resulting in prompt cytoplasmic transport of the vesicle’s contents [40]. This fusion process is especially important for cationic liposomes or those designed with fusogenic lipids like phosphatidylethanolamine, which stimulate the production of non-lamellar phases that aid in membrane coalescence. This technique is helpful for the transport of nucleic acids, peptides, and other intracellularly active substances since it avoids the endolysosomal breakdown pathway. In addition, lipid transfer is a subtle mechanism in which lipids from the nanoliposome integrate into the cell membrane, gradually altering its composition and facilitating controlled release without vesicle disruption. Similarly, stable adsorption occurs when nanoliposomes adhere to the cell surface through physicochemical interactions, creating localized reservoirs from which the bioactive compound is released slowly by diffusion or enzymatic action.

Stimuli-sensitive liposomes are used in advanced formulations and respond to specific environmental triggers such as pH, redox potential, enzymatic activity, temperature, or light [37]. These systems are intended to remain stable under normal physiological settings, releasing the cargo compound only when subjected to the desired stimuli. For example, PEGylated liposomes provide longer circulation time and lower immune clearance, although PEGylation may diminish membrane fusion capacity, encouraging endocytic absorption instead [41]. Targeted liposomes, such as immunoliposomes, contain ligands or antibodies released at targeted sites, increasing cellular absorption and therapeutic efficacy [42].

Specifically, in food applications, release mechanisms are designed to maintain gastrointestinal stability and intestinal absorption. Liposomes must endure stomach acidity and enzymatic destruction before releasing bioactive compounds in the intestine, which is commonly accomplished through contact with bile salts or enzymatic hydrolysis by pancreatic lipases. High cholesterol content and saturated fats increase stability, whereas cationic surface charge improves mucosal adherence and absorption.

## 3. Biological Activity of the Encapsulated Compounds

Encapsulation within liposomes helps preserve bioactive compounds’ structural integrity and functional properties by protecting them from degradation. This protective mechanism makes liposomal encapsulation a highly effective strategy for incorporating bioactives into foods to prevent or treat chronic degenerative diseases and various clinical conditions.

Carotenoids are colored natural pigments belonging to a large family of C_40_ skeleton with eight isoprene molecules. They are classified into xanthophylls and carotenes, with the former, such as lutein, β-cryptoxanthin, and astaxanthin, containing one or more oxygen atoms, while the latter, such as α-carotene and β-carotene, lycopene, and phytoene, consist of hydrogen and carbon atoms [43]. Carotenoid-rich foods have received significant attention in human health due to their physiological functions, including antioxidant and anticancer, and the ability to prevent chronic diseases such as age-associated macular degeneration and cardiovascular disease [44,45]. It has been well demonstrated that the functional properties of carotenoids were associated with their chemical structure, i.e., the number of conjugated double bonds and the presence of different kinds of end groups. However, these structural properties are also responsible for the carotenoid’s instability to light, high temperature, oxygen, and metal ions, resulting in high susceptibility to oxidation and low bioavailability [45]. Given the multiple health benefits of carotenoids, they are widely used as a natural colorant and antioxidant in pharmaceutical and food industries to prolong shelf life in dairy, meat, confectionery, and beverage products. However, carotenoids may undergo loss of functional properties during food processing owing to their instability and interaction with other food ingredients. Also, digestive enzymes and other nutrients in vivo and pH can alter carotenoid stability [44].

Astaxanthin (AST) is a natural carotenoid pigment widely found in plants, crustacean shells, flamingo feathers, and micro-organisms. Due to its various biological activities, AST has been suggested as an important compound in biochemical research. It has great potential for application in cosmetics, human nutritional health products, and medicines. Unfortunately, the poor water solubility, chemical instability, and low oral bioavailability make applying AST in food systems challenging. Besides, the amount of natural AST is limited, and how to extract and utilize AST efficiently is in great demand [46]. AST is a non-vitamin-A-derived, ketone-lipid soluble carotenoid [47], which presents a red-orange color and widely exists in many crustacean animals such as shrimp and crabs. It is also the highest-level product of carotenoid oxygen-containing derivatives [48]. The molecular structure of natural AST contains C=C double chain conjugated olefin structure. This structure’s specificity allows it to extinguish reactive oxygen species and scavenge free radicals effectively.

As a member of the liposoluble carotenoid family, the unique structure of AST may cause the following disadvantages regarding application properties. (1) Hydrophobicity: AST has a very poor solubility in nonpolar solvents as a lipophilic substance. Since there are two hydroxyl groups at each end of AST, each links one hydroxyl group that may interact with fatty acids to form esters. Esterified AST has a stronger hydrophobicity than free AST [45]. (2) Instability: The AST monomers are extremely unstable because of the structures of unsaturated conjugated double bonds. During processing and storage, they are easily degraded and fade under changes in light, temperature, and oxygen content, resulting in further loss of their original biological activity and poor quality and color of final products [47]. The above properties of AST may restrict its application. The effective design of the delivery system increases AST’s dispersibility and stability, avoids shortcomings, and increases its processing adaptability in the food industry.

Besides, carotenoids can improve human health, and other compounds such as phenols, alkaloids, nitrogen, and organosulfur compounds can significantly benefit human health. Phenolic compounds are considered secondary metabolites with various structures produced by plants. Structurally, phenolic compounds present a benzene ring (C6) with one or more hydroxyl (-OH) groups (s), including other functional substituents (glycosides, methyl ethers or esters) [49].

There are two metabolic pathways through which phenols can be produced in plants: the shikimic acid and the acetic acid pathways. The first produces polyphenols, and the second produces simple phenols. Combining these two pathways produces flavonoids, plants’ most plentiful group of phenolic compounds [50]. Phenolic compounds can be classified in various ways because they consist of various heterogeneous structures, ranging from simple to highly polymerized compounds. Based on this, they have been classified into three categories: shortly distributed (e.g., phenols, pyrocatechol, and hydroquinone), widely distributed (e.g., flavonoids and their derivatives, coumarins and phenolic acids), and polymers (e.g., tannin and lignin) [51].

According to their chemical structure, they can also be classified as soluble (e.g., phenol, flavonoids, and low- or medium-molecular-weight tannins) and insoluble (e.g., condensed tannins and phenolic acids). The first group is not bound to cell membrane compounds, while the second group is bound to cell wall polysaccharides or proteins. This classification is of great importance from a nutritional perspective because the digestion, absorption, and utilization of these compounds largely depend on their solubility. The main interest in phenolic compounds lies in their antioxidant activity, which is associated with beneficial health effects and the prevention of certain diseases. Additionally, they are also used therapeutically for their pharmacological properties. This property entirely depends on the chemical structure, as it may or may not have double bonds or molecules with resonance capacity. Among the phenolic compounds with known high antioxidant activity are flavonoids and tannins [51]. Table 2 presents the structures of the main flavonoids with antioxidant activity.

It has been determined that the high antioxidant capacity of flavonoids is due to their structural configuration, mainly the presence of the hydroxyl group (-OH) in the 3′ and 4′ positions of the B ring (intermediate), which also confers stability to the compound when it is transformed into a radical by electron donation. This activity is enhanced by the position of the double bonds in the 2 and 3 carbons of the C ring, together with the carbonyl group in the fourth position, allowing for the electrons’ movement between the benzene rings [52].

On the other hand, tannins can be classified into two major groups: hydrolyzable tannins and non-hydrolyzable tannins, or proanthocyanidins. Hydrolyzable tannins have a glucose center or polyhydric alcohol partially or completely esterified with gallic acid or hexahydroxydiphenic acid, forming gallotannins and ellagitannins, respectively [52].

**Figure 4:**
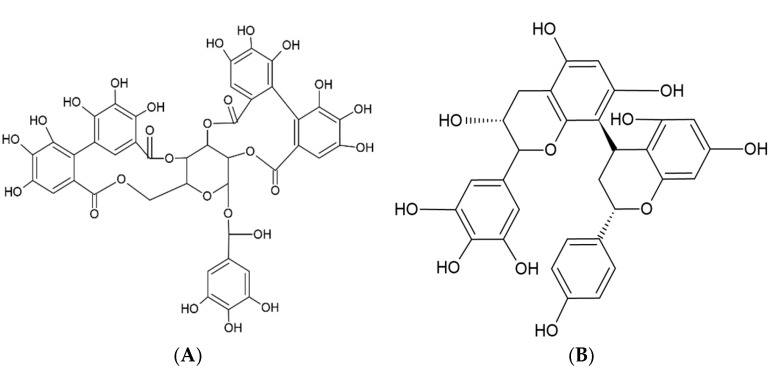
Chemical structures of a hydrolyzable tannin (**A**) and a non-hydrolyzable or proanthocyanidin tannin (**B**).

Proanthocyanidins are polymers of catechins and/or leucoanthocyanidins that are not hydrolyzable by acid treatment and are responsible for the astringent properties of plants. They are called proanthocyanidins because of their ability to transform into anthocyanidins. Although the antioxidant capacity of tannins has not been extensively exploited in the food industry, their activity is known to depend on the degree of polymerization of their chemical structures [53]. Figure 4 shows the structure of a hydrolyzable tannin and a non-hydrolyzable tannin.

### 3.1. Bioactive Compounds Encapsulated in Nanoliposomes

The food industry uses liposomes to encapsulate bioactive compounds, primarily individual antioxidant molecules. Among the most studied are retinoids such as retinol, retinoic acid, and retinol ester [54,55], as well as carotenoids including α-carotene, β-carotene, γ-carotene, astaxanthin, violaxanthin, zeaxanthin, and β-cryptoxanthin [56,57,58,59,60]. Various phenolic compounds have also been successfully encapsulated in nanoliposomes, such as gallic acid, protocatechuic acid, caffeic acid, p-coumaric acid, and salicylic acid [49,61]. Additionally, vitamins A, B, C, D, E, and K have been widely explored [62,63,64]. Generally, these compounds have been studied due to their antioxidant and health-promoting properties. The chemical structures of these compounds and the type of nanoliposomes in which they were encapsulated are summarized in Table 3.

Several antioxidant compounds in Table 3 are already available in commercial liposomal formulations, particularly in the nutraceutical and cosmetic industries. For instance, liposomal vitamin C is widely marketed as an oral supplement with enhanced bioavailability. Similarly, liposomal formulations of vitamins A, D3, E, and K are commercially available as part of dietary supplements intended to support immune and bone health. Liposomal β-carotene, astaxanthin, lutein, and zeaxanthin are also found in marketed products, especially in formulations targeting eye health and antioxidant protection. In cosmetics, liposomal retinol and salicylic acid are used in topical applications to improve skin penetration and reduce irritation. While other phenolic and carotenoid compounds in the table, such as gallic acid or γ-carotene, have demonstrated potential in experimental liposomal systems, they are not yet commercially available in liposomal form.

The biological activity of compounds encapsulated in liposomes may not be altered. For example, the astaxanthin’s antioxidant capacity with alpha-tocopherol does not change after they are encapsulated in liposomes. However, it should be noted that nanoliposomes can produce a controlled release of their contents. This leads to different kinetics of the original compound in transport throughout the body. Compounds encapsulated in liposomes must be endocytosed to be transferred inside the cell [65], implying that transport across biological membranes will be subject to this process. This could contrast with the transport of the unencapsulated compound, which may be different (simple diffusion, facilitated diffusion, active transport) and will depend on its physicochemical properties and the biological mechanisms available for that compound.

Almost all the techniques involve the dissolution of phospholipids in an organic solvent, followed by removing the organic solvent later in the process. This prior dissolution, followed by removing organic solvent, is important for forming liposomes. The building blocks of liposomes are phospholipids and/or cholesterol. The critical micelle concentration of most commonly used phospholipids is in the nanomolar range, and the concentration of phospholipids used for liposome manufacturing is much above the critical micelle concentration. This, along with the three-dimensional cylinder-like shape of each phospholipid, leads to the formation of liposomes along with lipid aggregates when phospholipids, as such, are exposed to an aqueous environment.

**Table 3:** Examples of antioxidant compounds encapsulated in nanoliposomes and their chemical structure.

	Retinoids			
Name	Chemical Structure	Type of Nanoliposome	General Bioactivity	Reference
Retinol	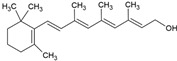	Lecithin-cholesterol structure, small unilamellar (20–200 nm), retinol is contained in the lipid intermembrane section	Not evaluated in the study Antioxidant, regulation of cell differentiation, epithelial and vision maintenance	[21,66]
Retinoic acid	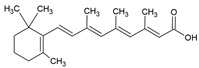	Lecithin-cholesterol structure, small unilamellar (20–200 nm), retinoic acid contained in the lipid intermembrane section	Not evaluated in the study Gene expression modulator, cell differentiation, antitumor activity, tissue regeneration	[66,67]
Retinyl ester	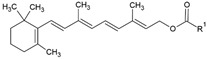	Lecithin-cholesterol structure, small unilamellar (20–200 nm), retinyl ester contained in the lipid intermembrane section	Not evaluated in the study Storehouse of vitamin A, precursor of active retinol	[66,67]
**Carotenoids**				
**Name**	**Chemical structure**	**Type of nanoliposome**	**General bioactivity**	**Reference**
α-carotene	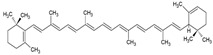	Lecithin, cholesterol, and polysorbate 80 structure, giant unilamellar size (>1 µm), α-carotene contained in the lipid intermembrane section	Not evaluated in the study Antioxidant, precursor of vitamin A, protector against oxidative stress	[21,68]
β-carotene	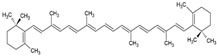	Lecithin, cholesterol, and polysorbate 80 structure, giant unilamellar size (>1 µm), β-carotene contained in the lipid intermembrane section	Not evaluated in the study Powerful antioxidant, precursor of vitamin A, protection against free radicals	[21,68]
γ-carotene	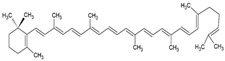	Soy, egg, or marine lecithin and cholesterol structure, giant unilamellar size (>1 µm), γ-carotene contained in the lipid intermembrane section	Not evaluated in the study Antioxidant, less active than β-carotene; contributes to cellular homeostasis	[21,69]
Astaxanthin	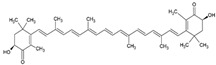	Agarose oligosaccharides, phosphatidylcholine, phosphatidyl galactose and/or phosphatidyl neoagarobiose structure, small unilamellar (20–200 nm), astaxanthin contained in the lipid intermembrane section	Potent antioxidant Not evaluated in the study: anti-inflammatory, photoprotective, cardiovascular, and ocular protector	[45,70]
Lutein	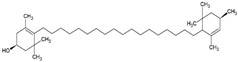	Supercritical carbon-dioxide method, small unilamellar size (20–200 nm), lutein contained in the aqueous center	Not evaluated in the study Antioxidant, protects the retina, eye photoprotector	[71]
Zeaxanthin	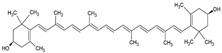	Lecithin, cholesterol, and polysorbate 80 structure, giant unilamellar size (>1 µm), zeaxanthin contained in the lipid intermembrane section	Not evaluated in the study Eye protection against blue light, antioxidant in retina	[45,68]
β-criptoxanthin	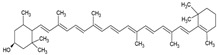	Cholesterol and phosphatidylcholine structure, small unilamellar (20–200 nm), β-cryptoxanthin contained in the aqueous center	Antioxidant	[58]
**Phenols**				
**Name**	**Chemical structure**	**Type of nanoliposome**	**General bioactivity**	**Reference**
Gallic acid	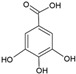	Soy lecithin and cholesterol structure, small unilamellar size (20–200 nm), gallic acid contained in the aqueous center	Antioxidant, antimicrobial	[72]
Protocatechuic acid	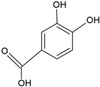	Egg yolk phosphatidylcholine and cholesterol structure, small unilamellar size (20–200 nm), and protocatechuic acid contained in the aqueous center	Antioxidant, cytoprotective effect against H_2_O_2_-induced cytotoxicity on mouse fibroblast cells	[73,74]
Caffeic acid	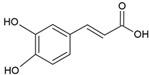	Egg yolk phosphatidylcholine and cholesterol structure, small unilamellar size (20–200 nm), and protocatechuic acid contained in the aqueous center	Antioxidant, cytoprotective effect against H_2_O_2_-induced cytotoxicity on mouse fibroblast cells	[73,74]
*p*-cumaric acid	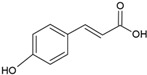	Soy lecithin and cholesterol structure, small unilamellar size (20–200 nm), *p*-coumaric acid contained in the aqueous center	Antimicrobial, antioxidant	[68]
Salicylic acid	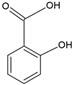	Soy lecithin and cholesterol structure, small unilamellar size (20–200 nm), *p*-coumaric acid contained in the lipid intermembrane section	Not evaluated in the study Anti-inflammatory, moderate antioxidant properties	[75]
**Vitamins**				
**Name**	**Chemical structure**	**Type of nanoliposome**	**General bioactivity**	**Reference**
Vitamin A	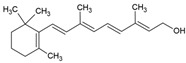	Lecithin and cholesterol structure, small unilamellar size (20–200 nm), and vitamin A contained in the lipid intermembrane section	Not evaluated in the study Antioxidant, immunomodulator, regulator of cell differentiation.	[66,67]
Vitamin B2	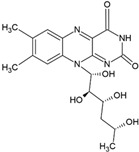	Vegetable oil (chia, sunflower, and virgin olive) structure, giant unilamellar size (>1 µm), vitamin B2 contained in the lipid intermembrane section	Not evaluated in the study Enzyme cofactor, antioxidant, participation in energy metabolism	[67,76]
Vitamin C	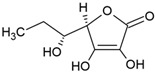	Phosphatidylcholine, stearic acid, and stearic calcium structure, giant unilamellar (>1 µm), vitamin C contained in the aqueous center	Not evaluated in the study Neutralization of free radicals in aqueous media, collagen synthesis, absorption of non-heme iron, stimulation of the immune system	[3,21]
Vitamin D3	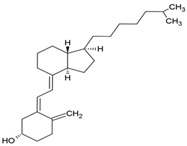	Soy phosphatidylcholine and cholesterol structure, giant unilamellar size (>1 µm), vitamin D3 contained in the lipid intermembrane section	Not evaluated in the study Regulates calcium and phosphorus homeostasis, immunomodulator, essential for bone and immune health	[67,77]
Vitamin E	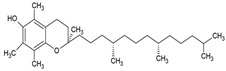	Phosphatidylcholine, stearic acid, and stearic calcium structure, giant unilamellar (>1 µm), vitamin E contained in the aqueous center	Not evaluated in the study Protection of cell membranes against oxidative stress, prevention of lipid peroxidation	[67]
Vitamin K	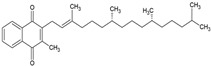	Phosphatidylcholine and cholesterol structure, giant unilamellar size (>1 µm), vitamin K contained in the lipid intermembrane section	Not evaluated in the study Activation of proteins involved in blood coagulation, bone metabolism, and osteoporosis prevention	[67,78]

To make uniform liposomal dispersions, it is important to make thin lipid sheets before exposing them to an aqueous phase or introducing the organic phospholipid solution in a controlled manner in an aqueous environment for the formation of liposomes. This is why all the reported techniques of liposome manufacturing—i.e., solvent evaporation, solvent dispersion/antisolvent addition, or detergent removal—focus on first disaggregating the phospholipids into individual phospholipid molecules, followed by exposure to an aqueous environment to enable the formation of different types of liposomes [79].

### 3.2. Carotenoids Encapsulated in Nanocarriers

**Figure 5:**
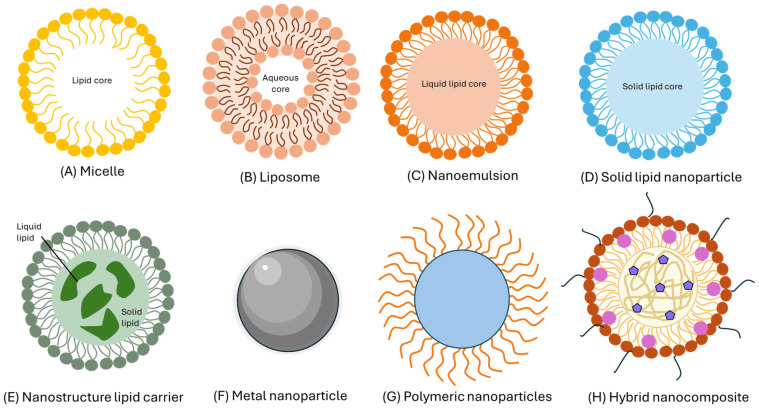
Representative nanosystems for the delivery of bioactive compounds. Each system differs in composition and structure, enabling the encapsulation, protection, and controlled release of hydrophilic or lipophilic compounds depending on the application.

Thus, transforming from microencapsulation to nanoencapsulation plays a pivotal role in reducing particles to nanosize by employing either top-down or bottom-up methods [80]. Recent advancements in the field of nanoscience and nanotechnology have enabled the preparation of nanoscale functional compounds by encapsulating them into a wide variety of nanostructures, including nanoemulsions (NEs), nanoliposomes (NLs), nanocapsules (NCs), nanofibers (NFs), nanoparticles (NPs), solid lipid nanoparticles (SLNPs), nanostructured lipid carriers (NLCs), and supercritical fluid-based nanoparticles (Figure 5) [81].

Due to the increasing prevalence rate of chronic diseases, the emerging challenges in delivering functional compounds to target tissues, organs, and cells, as well as instability, poor aqueous solubility, bioavailability, and low release and absorption in vivo, cannot be overcome by microencapsulation techniques. Recent developments in the field of nanotechnology have provided some excellent means to reduce particle size through top-down (high-energy method) or bottom-up (self-assembly) processes [82]. Such a reduction in particle size has enhanced the stability, targeting ability, bioavailability, and release properties [83]. Most importantly, the reduction in particle size enables penetration into deeper portions of cells or tissues, resulting in high bioavailability [7].

Research studies published within the last five years on nanoencapsulation of various carotenoid compounds mention that it can be done by using different preparation techniques. These studies demonstrated the impact of nanoencapsulation on improving physicochemical properties, bioavailability, controlled release, and bioactivity. Table 4 summarizes various nanosystems used to encapsulate carotenoids. Table 5 highlights the advantages and disadvantages of some nanosystems and explores the main cell uptake and the intracellular traffic. Most nanocarriers, including nanoliposomes, nanoemulsions, SLNPs, NLCs, and metal nanoparticles, enter cells primarily through endocytosis mechanisms, including clathrin-mediated endocytosis, caveolin-mediated endocytosis, clathrin/caveolin-independent endocytosis, phagocytosis (especially in immune cells), and micropinocytosis. The specific pathway depends on the physicochemical characteristics of the nanoparticle (size, charge, surface composition) and the cell type [83]. After internalization, most nanocarriers are confined in endocytic vesicles (early endosomes), where the pH is moderately acidic. These vesicles can fuse with lysosomes, where the acidic environment and enzymes can degrade the nanocarriers and release their contents. In addition, they can undergo recycling to the plasma membrane (recycling endosomes), limiting effective intracellular delivery, or mature into late and multivesicular endosomes, facilitating the release of cargo into the cytoplasm if the nanoparticle manages to escape the endosomal compartment. Endosomal escape is critical for effectively delivering bioactives that require cytosolic or nuclear action. Depending on the composition and functionalization, nanocarriers can be targeted to different organelles, such as the cytoplasm, the nucleus, mitochondria, Golgi apparatus, or endoplasmic reticulum, if the nanoparticle is functionalized for specific recognition or exocytosis which part of the nanocarrier may be expelled back into the cell, limiting delivery efficiency [84,85].

## 4. Nanoliposomes Enhance Foods and Human Health

In recent years, the food industry has played a prominent role in developing functional foods, which provide health benefits beyond the essential nutritional value inherent in the food. These foods contain bioactive components, either chemical compounds naturally present in the food or formed and added during processing, which can exert specific biochemical and physiological functions when consumed by humans [116]. For example, particular lipids in milk have recognized biological properties; conjugated linoleic acid (CLA) can be mentioned. It is a generic term used to describe the mixture of positional and geometric isomers of linoleic acid (C18:2 9c12c) with conjugated double bonds. In recent years, they have gained considerable attention, as it is believed that some of these isomers (C18:2 9c, 11t and C18:2 10t, 12c) have beneficial biological effects (reduction of body fat content and increase in muscle mass, stimulation of the immune system, among others) [117].

The deterioration of CLA, primarily through oxidation, leads to a decrease in its concentration, loss of bioactivity, and the appearance of unwanted molecules that negatively impact the nutritional and sensory quality of the food. An approach to achieve dairy products enriched in this bioactive compound with good characteristics, without the indicated adverse effects, is the addition of CLA protected by encapsulation, which constitutes a promising alternative. Among encapsulation methods, a very innovative strategy to preserve pharmaceutical or food compounds is that of liposomes [117].

In a previous study, ref. [99] evaluated the impact of adding a lyophilized powder of liposomes with conjugated linoleic acid (NL-CLA) during yogurt production. They prepared yogurts with CLA in liposomes and controlled yogurts without CLA. They determined the stability of the fatty acid during storage (21 days at 4 °C) and the parameters—pH, acidity, syneresis, microbiological counts (total lactic acid bacteria, molds, and yeasts, total aerobic mesophilic germs), dry residue, fat, and protein content—using standardized techniques. Additionally, they observed the microstructure of the yogurts. Adding nanoliposomal vehicles loaded with CLA did not modify the fermentation time; at the end of maturation, the pH and acidity (°D) values remained within appropriate ranges for all yogurts: 4.3–4.4 and 96–99, respectively. The lactic acid bacteria count of the starter reached 10^9^ CFU/g, and no contaminating micro-organisms were detected. The total solids, protein, and fat contents showed typical values. At the end of storage, yogurts with liposomes exhibited lower syneresis than the controls.

These results correlated with microstructure observations, showing a modification in the protein matrix. The CLA content increased successfully in yogurts with the addition of the liposomal ingredient, as the basal amount of CLA tripled. Thus, the feasibility of applying an ingredient rich in bioactive lipids in the yogurt food matrix was verified.

With the aforementioned information and based on various studies, it can be confirmed that the addition of liposomes to food improves its characteristics and antioxidant properties. All of this is due to minimal or no modification of the basic structures and the prolonged release of the compounds encapsulated in the liposomes.

Introducing nanoliposome-enhanced foods has sparked great intrigue in society, as they are believed to improve human health through antioxidant, antihemolytic, antimicrobial, anti-inflammatory, photoprotective, and/or anticancer activity and prolonged liberation properties. Innovative encapsulation techniques are currently applied to protect the structure and function of food compounds and nutraceutical properties and improve their bioavailability. Nano-vehicles or nanocarriers offer the food processor several advantages by ensuring against nutritional loss and incorporating time-release mechanisms into the formulation [37].

The development and application of nanoliposomes in food technology and the food industry are considerably less advanced compared to their use in the pharmaceutical and cosmetic sectors [96]. However, it is speculated that the greatest advantages will be seen in the agriculture and dairy industries. The limited development is due to the challenges in finding safe, low-cost methods for production in the shortest possible time to produce liposomes on a large scale. Studies to date indicate that the potential of nanoliposomes in food lies in their ability to enhance flavor or in accelerated maturation techniques (e.g., cheese maturation), prolonged and targeted release, the synergistic activity of different encapsulated antioxidant compounds, and the stabilization of minerals, such as calcium and iron in dairy products [37]. The ability of nanoliposomes to provide targeted delivery of the encapsulated material in specific areas of the food system is highly beneficial for the dairy industry. For example, employing proteinase enzymes encapsulated in the lipid vesicles can significantly reduce the time and cost of cheese ripening.

Besides the previous statement, cheese can be improved in its nutritional properties by adding vitamins C, D, and E, not necessarily in co-encapsulation. However, they can interact with each other once released. These vitamins have many properties, including helping protect cells from damage caused by free radicals, collagen formation (necessary for the formation of this protein used to produce skin, tendons, ligaments, and blood vessels), strengthening the immune system by helping to keep the immune system strong and reduce the chances of getting sick, regeneration of vitamin E, body calcium absorption (a mineral essential for the formation of strong bones), and reducing the risk of cancer, cardiovascular disease, depression, and autoimmune diseases [118,119].

Otherwise, minerals can also be encapsulated in nanocarriers to avoid, principally, iron deficiency, which causes the risk of developing anemia. This condition is mainly due to insufficient dietary intake of iron, lack of bioavailability, or both. Iron shortage in blood should not be ignored as it may cause anemia when the hemoglobin level falls below standard levels, a disease that, if not treated, can evolve into leukemia [120]. Hence, enriching dairy products with iron to evade fat oxidation and metallic off-flavor is necessary. To achieve this, supplementation of dairy products with iron encapsulated in safe and nontoxic carriers has been developed. Towards this end, a highly soluble iron (i.e., ferrous sulfate) is preferred due to its cost-effectiveness and high bioavailability [37]. Another example of a mineral is magnesium. It is an essential mineral compound that is associated with lowering the risk of some clinical disorders, including cardiovascular disease, hypertension, type 2 diabetes, and muscular weakness [121].

## 5. Conclusions

Nanoliposomes offer significant advantages by protecting encapsulated compounds from degradation by environmental and enzymatic factors, enabling controlled and targeted release in the body. Furthermore, their structural versatility allows for encapsulating hydrophilic and lipophilic compounds, broadening the range of applications in the food, nutraceutical, and pharmaceutical industries. However, challenges remain related to optimizing encapsulation efficiency, storage stability, and site-specific release, which must be addressed for their large-scale implementation. The use of various types of phospholipids increases the range of compounds that can be encapsulated, making this not only an innovative application but also one with a trend of expansion into various scientific fields, such as healthcare, food industries, cosmetics, and pharmaceuticals.

## 6. Prospects

Prospects for the development and application of nanoliposomes in the field of antioxidants are promising. Research on the encapsulation of complex extracts rich in pigments and polyphenols is anticipated, as is the integration of innovative release technologies that respond to specific physiological stimuli. Furthermore, the trend toward functional and personalized foods will drive innovation in nanoencapsulation systems tailored to different health needs and population groups. Long-term clinical and safety studies will be essential, as will the development of clear regulations for their use in food and nutraceutical products. Finally, interdisciplinary collaboration between food scientists, chemists, biotechnologists, and healthcare professionals will be key to translating these technological advances into tangible benefits for public health.

## Figures and Tables

**Table 2:** Chemical structures of the principal classes of flavonoids.

Flavonoid	Structure
Flavones	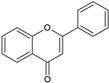
Flavonols	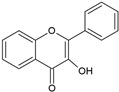
Flavanones	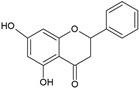
Flavanols	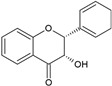
Anthocyanidins	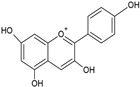
Isoflavones	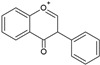

**Table 4:** Nanosystems for encapsulation of carotenoids. Obtained and edited from Rehman et al. [81].

Nanosystem	Carotenoids	Particle Size (nm)	EE (%)	Zeta Potential (mV)	Storage Stability (Days)	References
Nanoemulsions	β-carotene	218	NA	40	21 at 37 °C	[86]
		143.7		−38.2	30 at 25 °C	
	Microbial carotenoids	142.1		NA	30 at 25 °C	[86]
	Carotenoids	290 to 350		−53.4 to −58.8	21 at 25 °C	[87]
	β-carotene	198.4 to 315.6		−29.9 to −38.5	90 at 4, 25, and 37 °C	[88]
	Carotenoids	<200		−30 to −45	35 at 25 °C	[89]
	Lycopene	145.1 to 161.9		−19.7 to −20.7	1 at 25 °C	[90]
		200.1 to 287.1	61 to 89.1	20 to 45	42 at 4, 25, and 37 °C	[91]
Polymeric/biopolymeric NPs	Carotenoids	153	83.7	NA	NA	[92]
		84.4	>96	−41.3 to −43.6	60 at 41 °C	[93]
	β-carotene	77.8 to 371.8	98.7 to 99.1	−37.8 to −29.9	NA	[94]
	β-carotene	70.4	97.4	NA	NA	[59]
	Lycopene	152	89	58.3	NA	[95]
		~200	>95	−36	210 at 5 °C	[96]
		193	NA	−11.5	14 at 25 °C	[97]
	Lutein	<250	74.5	−27.2	NA	[98]
	Lutein	240 to 340	~91.9	NA	NA	[99]
	Crocetin	288 to 584	59.6 to 97.2	NA	NA	[100]
	Fucoxanthin	200 to 500	47 to 90	30 to 50	6 at 37 °C	[101]
Nanoliposomes/liposomes	Carotenoids	70 to 100	75	−5.3	NA	[102]
	β-carotene	162.8 to 365.8	~98	64.5 to 42.6	70 at 4 °C	[103]
	Astaxanthin	80.6	97.6	31.8	15 at 4 and 25 °C	[104]
		60 to 80	97.4	NA	NA	[105]
	Lutein	264.8 to 367.1	91.8 to 92.9	−34.3 to −27.9	NA	[62]
SLNPs and NLCs	β-carotene SLNPs	200 to 400	53.4 to 68.3	−6.1 to −9.3	90 at 5, 25, and 40 °C	[106]
		<220	NA	20 to 30	10 at 25 °C	[106]
		120	NA	−30	56 at 25 °C	
	Lycopene SLNPs	125 to 166	86.6 to 98.4	NA	60 at 4 °C	[107]
	Lycopene NLCs	157 to 166	> 99	−74.2 to −74.6	120 at 4, 30, and 40 °C	[79]
		121.9	84.50	−29	90 at 25 °C	[7]
Supercritical fluid-based NPs	Astaxanthin	150 to 175	NA	NA	NA	[65]
		266	84	NA	NA	[108]
Metal/metal oxide-based NPs and hybrid nanocomposites	Carotenoids	20 to 140	NA	NA	NA	[109]
	Lycopene	3 to 5		−48.5	90 at 4 and 25 °C	[110]
		20.8		−25.3	NA	[111]

EE = encapsulation efficiency, NPs = nanoparticles, SLNPs = solid lipid nanoparticles, NA = data not available and NLCs = nanostructured lipid carriers.

**Table 5:** The advantages and disadvantages of nanosystems for encapsulation of carotenoids. Obtained and edited from [72].

Nanosystem	Advantages	Disadvantages	Main Physiological Phenomena	References
Nanoemulsions	High optical clarity and enhanced physical stability Small-sized particles with improved bioavailability and absorption Increased solubility of lipophilic compounds Rapid and efficient penetration of the compound Energy-efficient method	Use of large surfactant and co-surfactant Low storage and chemical stability Limited solubility for high melting substances Biotoxicity of the carrier	Internalized mainly by clathrin- or caveolin-mediated endocytosis. Intracellular trafficking: endosomal escape.	[43,57,112]
Polymeric/biopolymeric NPs	High stability and EE Easy biodegradability and high bioavailability Controlled release, drug targeting, and enhanced cellular uptake Low cost	Irritation after administration Low storage stability	NPs entry into cell using different endocytotic pathways: macropinocytosis. phagocytosis, clathrin-mediated endocytosis, clathrin-caveolin independent endocytosis, and caveolae-mediated endocytosis. Intracellular trafficking: endosomal escape	[43,57,83]
Nanoliposomes/liposomes	Less toxicity Increased stability, efficiency, and pharmacokinetic effects	Low solubility, short half-life, and low EE Difficult to control the size of liposomes Less reproducibility High-cost ingredients Poor resistance to gastrointestinal enzymes at low pH	Fusion with cell membranes or endocytosis; intracellular trafficking to lysosomes or cytosol	[57,80,83]
SLNPs	High possibility of encapsulating lipophilic and hydrophilic compounds No use of organic solvents Easy scale-up process High membrane permeability of liposomes and the ability of biopolymer NPs for controlled release High bioactive absorption and easy biodegradability Lack of biotoxicity	Low EE and stability The presence of other colloidal structures Polymorphic transitions may result in the expulsion of bioactive compounds Conformational modification of the lipid NPs	Internalized mainly by clathrin- or caveolin-mediated endocytosis. Intracellular trafficking: endosomal escape, lysosome	[43,57,80,85]
NLCs	High EE and stability Controlled release Simple preparation methods with controlled particle size High possibility for scale-up	Cytotoxic effect The irritation and sensitizing action of surfactants	Internalized mainly by clathrin- or caveolin-mediated endocytosis. Intracellular trafficking: endosomal escape, lysosome	[57,80,85]
Supercritical fluid-based NPs	Scalable, green, nontoxic, and economical Good particle size with controlled particle morphology High production yield and EE Homogeneous drug distribution Reduced isomerization and thermal degradation of heat-labile compounds Solvent can be easily eliminated from the food matrix Minimizes harmful chemical residues Low-temperature operation Produces solvent-free and homogeneous products Single-step processing method	Poor solubility of solutes in SCF CO_2_ The size of particles cannot be controlled	Internalized mainly by clathrin- or caveolin-mediated endocytosis. Intracellular trafficking: endosomal escape, lysosome	[43,80,84,113]
Metal/metal oxide-based NPs and hybrid nanocomposites	No toxic solvent required Great plasma absorption Target site delivery High surface area Cost-effective High uniformity in shape, size, and branch length	Particles instability Toxic, carcinogenic, and causes irritation Less reproducibility of the processes Low possibility for scale-up	Internalized mainly by clathrin- or caveolin-mediated endocytosis. Intracellular trafficking to lysosomes	[84,114,115]

EE = encapsulation efficiency; NPs = nanoparticles; SLNPs = solid lipid nanoparticles; and NLCs = nanostructured lipid carriers.

## Data Availability

No new data were created or analyzed in this study. Data sharing is not applicable to this article.

## Abbreviations

AST	Astaxanthin
CLA	Conjugated linoleic acid
DMPC	1,2-dimyristoyl-sn-glycero-3-phosphocholine
DPPG	1,2-Dipalmitoyl-sn-glycero-3-phosphoglycerol
DSPC	1,2-Distearoyl-sn-glycero-3-phosphocholine
DSPG	1,2-Distearoyl-sn-glycero-3-phosphoglycerol
EE	Encapsulation efficiency
NA	Data not available
NCs	Nanocapsules
NEs	Nanoemulsions
NFs	Nanofibers
NLCs	Nanostructured lipid carriers
NLs	Nanoliposomes
NPs	Nanoparticles
PA	Phosphatidic acid
PC	Phosphatidylcholine
PG	Phosphatidyl-glycerol
PI	Phosphatidylinositol
PS	Phosphatidylserine
SLNPs	Solid lipid nanoparticles
